# Serum BDNF in subacute stroke: effects of a four-week aerobic exercise intervention in the PHYS-STROKE trial

**DOI:** 10.3389/fstro.2026.1897859

**Published:** 2026-09-15

**Authors:** Torsten Rackoll, Lea Doppelbauer, Konrad Neumann, Alexander Heinrich Nave

**Affiliations:** 1 Department for Neurology with Experimental Neurology, Charité—University Medical Center Berlin, corporate member of Freie Universität Berlin and Humboldt–Universität zu Berlin, Berlin, Germany; 2 QUEST Center for Responsible Research, Berlin Institute of Health at Charité—University Medical Center Berlin, Berlin, Germany; 3 German Center for Cardiovascular Disease (DZHK), Partner Site Berlin, Berlin, Germany; 4 Department for Gynecology with Breast Center, Charité—University Medical Center Berlin, corporate member of Freie Universität Berlin and Humboldt–Universität zu Berlin, Berlin, Germany; 5 Institute of Biometry and Clinical Epidemiology, Charité—University Medical Center Berlin, corporate member of Freie Universität Berlin and Humboldt-Universität zu Berlin, Berlin, Germany; 6 Center for Stroke Research Berlin, Charité—University Medical Center Berlin, corporate member of Freie Universität Berlin and Humboldt–Universität zu Berlin, Berlin, Germany

**Keywords:** aerobic exercise, brain-derived neurotrophic factor, BDNF, neuroplasticity, stroke recovery, subacute stroke

## Abstract

**Introduction:**

The early phase after stroke is considered a period of heightened neuroplasticity and may be responsive to rehabilitation interventions targeting biological recovery. Brain-derived neurotrophic factor is one candidate mediator of post-stroke plasticity, but it remains unclear whether clinically feasible aerobic exercise induces sustained increases in circulating brain-derived neurotrophic factor after stroke.

**Methods:**

We performed a pre-specified secondary biomarker analysis of the multicentre, randomized, endpoint-blinded Physical Fitness Training in Patients with Subacute Stroke trial. Participants were randomized to 4 weeks of moderate-intensity treadmill-based aerobic training or relaxation therapy. Serum brain-derived neurotrophic factor was assessed from baseline to 6 months post-stroke. Models were adjusted for baseline brain-derived neurotrophic factor, age, sex, stroke severity, platelet count, depressive symptoms, smoking status, and study center.

**Results:**

Among 200 randomized participants, serum brain-derived neurotrophic factor increased modestly from baseline to 3 months and remained stable thereafter. No relevant between-group difference was observed at 6 months after aerobic training compared with relaxation therapy (training: 24.6 ng/mL; 95% CI, 20.2–29.0 vs. relaxation: 24.3 ng/mL; 95% CI, 19.8–28.8; adjusted *p* = 0.74). Findings were consistent in complete-case and change-score sensitivity analyses. Exploratory analyses showed no robust treatment effect modification by depressive symptoms or intervention dose. A nominal sex-related subgroup finding should be interpreted cautiously.

**Discussion:**

Moderate-intensity aerobic exercise in early subacute stroke rehabilitation was not associated with sustained serum brain-derived neurotrophic factor increases compared with relaxation therapy. These findings argue against a robust long-term peripheral neurotrophin response, while not excluding acute, higher-intensity, or subgroup-specific effects.

**Clinical Trial Registration:**

[clinicaltrials.gov/study/NCT01953549], identifier [NCT01953549].

## Introduction

The acute and early subacute phases after stroke represent a period of heightened biological responsiveness, during which spontaneous recovery and experience-dependent neuroplasticity contribute to functional improvement. Recent clinical evidence suggests that rehabilitation may be particularly effective during the first 2–4 months after stroke, although recovery can continue beyond this period and the duration of the sensitive window varies between individuals. This early phase therefore provides an important opportunity to investigate biological mechanisms that may influence responsiveness to rehabilitation (Dromerick et al., 2021; Li, 2023; Li et al., 2024).

Among the molecular mediators implicated in post-stroke neuroplasticity, brain-derived neurotrophic factor (BDNF) has received particular attention (Ashcroft et al., 2022). BDNF supports neuronal survival, synaptogenesis, dendritic spine formation, and long-term potentiation, primarily through activation of the TrkB receptor and associated intracellular signaling pathways (Reichardt, 2006). Experimental models consistently demonstrate that increased BDNF expression is associated with enhanced motor recovery after stroke, providing a biologically plausible rationale for targeting BDNF through exercise-based interventions (Mang et al., 2013).

Aerobic exercise is widely recommended in subacute stroke rehabilitation by international guidelines, yet the biological mechanisms through which exercise may influence recovery during this phase remain incompletely understood (Rasmussen et al., 2009; Dinoff et al., 2016; MacKay-Lyons et al., 2020; Billinger et al., 2014). In humans, aerobic exercise reliably increases circulating BDNF concentrations in healthy individuals (Müller et al., 2020). Increased serum BDNF levels after exercise are assumed to be released primarily from the brain (Rasmussen et al., 2009). In contrast, evidence in people with stroke remains inconsistent. Serum BDNF levels after stroke are generally lower than in healthy reference populations and have been associated with stroke severity and long-term functional outcome (Mojtabavi et al., 2022). However, some randomized trials examining the effects of aerobic exercise on peripheral BDNF during the subacute phase have reported small or absent effects, alongside substantial inter-individual variability. Recent evidence indicates that exercise-related changes in circulating BDNF after stroke are heterogeneous and may depend on exercise intensity, timing, modality, and intervention duration (Ashcroft et al., 2022; Limaye et al., 2021; Alcantara et al., 2018). A systematic review and meta-analysis suggested more consistent BDNF increases after high-intensity aerobic exercise, whereas effects of low- and moderate-intensity exercise were smaller and less consistent (Ashcroft et al., 2022). Studies in subacute and chronic stroke populations have likewise reported absent or variable serum BDNF responses after moderate-intensity aerobic exercise (Maguire et al., 2023). Acute responses should be distinguished from sustained changes, as transient increases immediately after exercise may not persist during longer-term follow-up. High-intensity interval exercise may elicit stronger acute peripheral BDNF responses than moderate-intensity continuous exercise, although evidence for persistent effects remains limited (Rodríguez-Gutiérrez et al., 2024).

Interpretation of peripheral BDNF measurements requires caution. Serum BDNF is strongly influenced by platelet storage and release and is modulated by metabolic, inflammatory, neuroendocrine, and pharmacologic factors (Gejl et al., 2019). While serum BDNF cannot be considered a direct surrogate of central nervous system BDNF signaling, it is commonly used as a peripheral marker of BDNF-related biological processes with potential relevance to neuroplasticity (Rasmussen et al., 2009; Boukhatem et al., 2024). From this perspective, variability in serum BDNF responses may provide insight into differential biological responses to rehabilitation interventions across patient subgroups rather than reflecting uniform treatment effects.

The multicentre randomized Physical Fitness Training in Patients with Subacute Stroke (PHYS-STROKE) trial compared a 4-week programme of moderate-intensity treadmill-based aerobic training with an active control intervention based on relaxation therapy in patients in the early subacute phase after stroke. The intervention was designed as a pragmatic, safety-oriented aerobic training programme for moderately to severely affected patients in early subacute inpatient rehabilitation. The primary clinical outcomes of the trial have been reported previously (Nave et al., 2019). In addition to functional outcomes, serum BDNF were measured longitudinally from baseline up to 6 months after stroke (Nave et al., 2013).

In this pre-specified secondary analysis, we examined whether moderate-intensity aerobic training was associated with differences in serum BDNF trajectories compared with relaxation therapy, with particular attention to the immediate post-intervention period and 6-month follow-up. Second, recognizing the heterogeneity of post-stroke recovery, we explored whether relevant baseline characteristics modified associations between treatment allocation and peripheral BDNF responses. We hypothesized that aerobic training increases serum BDNF at 6 months compared to relaxation. These exploratory analyses were intended to generate hypotheses for stratified rehabilitation approaches rather than to establish causal mechanisms.

## Methods

### Study design

The present investigation is based on prospectively acquired data from the Physical Fitness Training in Patients with Subacute Stroke (PHYS-STROKE) trial (Nave et al., 2019). PHYS-STROKE was a multicentre, assessor-blinded, randomized controlled trial designed to compare 4 weeks of treadmill-based aerobic exercise with relaxation therapy as an active control intervention in individuals 5–45 days after stroke. Recruitment took place between 26 September 2013 and 30 April 2017 across seven rehabilitation clinics and early-rehabilitation wards in the Berlin area, Germany. The study was registered at ClinicalTrials.gov (NCT01953549; registration date: 28 March 2013; clinicaltrials.gov/study/NCT01953549), and the primary efficacy outcomes have been reported previously. The original trial was conducted and reported in accordance with the CONSORT statement. The protocol received approval from the Ethics Committee of Charité—Universitätsmedizin Berlin (EA1/138/13). The study was conducted in accordance with the Declaration of Helsinki. All participants provided written informed consent prior to enrolment.

### Participants and randomization

Inclusion criteria comprised age ≥18 years, imaging-confirmed ischaemic or haemorrhagic stroke, and moderate to severe functional impairment defined as a Barthel Index score ≤ 65. Major exclusion criteria were progressive neurological deterioration, stroke caused by ruptured aneurysm or arteriovenous malformation, severe cardiovascular or neurological conditions preventing safe participation in exercise, substantial musculoskeletal restrictions, and severe psychiatric disorders. A comprehensive overview of eligibility criteria is provided in Table 1.

**Table 1:** Inclusion and exclusion criteria.

INCLUSION CRITERIA	
1.	Diagnosis of stroke (inclusion within 5–45 days after stroke onset); ischemic or haemorrhagic (cortical, subcortical, and brainstem), as determined by initial MRI/CT scan of the brain
2.	Age ≥ 18 years
3.	Able to sit unsupported (i.e., without holding onto supports such as the edge of the bed), with feet supported, for at least 30 s
4.	Barthel-Index ≤ 65 at inclusion
5.	Considered able to perform aerobic exercise, as determined by responsible physician
6.	Provision of written informed consent
EXCLUSION CRITERIA	
1.	Patient considered unable to comply with study requirements
2.	Stroke due to intracranial hemorrhage primarily due to bleeding from ruptured aneurysm or arteriovenous malformation
3.	Progressive stroke
4.	Unable to perform the required exercises due to (a) medical, (b) musculo-skeletal, or (c) neurological problems (for details see below, 4a–c)
4a.	Medical problems: unstable cardiovascular condition, or other serious cardiac conditions (e.g., New York Heart Association criteria for Class IV heart disease, hospitalization for myocardial infarction or heart surgery within 120 days, severe cardiomyopathy or documented serious and unstable cardiac arrhythmias)
4b.	Musculoskeletal problems: restricted passive range of motion in the major lower limb joints (i.e., an extension deficit of > 20° for the affected hip or knee joints, or a dorsiflexion deficit of > 20° for the affected ankle)
4c.	Neurological problems: severity of stroke-related deficits
5.	Required help of at least 1 person to walk before stroke due to neurological (e.g., advanced Parkinson's disease, Amyotrophic Lateral Sclerosis, Multiple Sclerosis) or non-neurological co-morbidities (e.g., heart failure, orthopedic problems)
6.	Life expectancy of less than 1 year as determined by responsible physician
7.	Drug or alcohol addiction within the last 6 months
8.	Significant current psychiatric illness defined as medication-refractory of bipolar affective disorder, psychosis, schizophrenia or suicidality.
9.	Current participation in another interventional trial

MRI, magnetic resonance imaging; CT, computer tomography

After completion of baseline assessments, participants were allocated in a 1:1 ratio stratified by age ( ≤ 65 vs. > 65 years), Functional Ambulation Category (FAC ≤ 3 vs. >3) and center to aerobic training or relaxation therapy *via* a secure web-based randomization system (Medical University of Graz, 2024). Outcome assessors, investigators and the trial statistician remained blinded of treatment assignment throughout the trial but not during this subanalysis.

### Interventions

Experimental interventions were delivered in addition to standard care which in Germany comprises daily physiotherapy, as well as occupational, speech and neuropsychological therapy depended on the patient's symptoms. Total therapy time was 50 min which included active training time and time for preparation, five times per week over a 4-week period with a target of up to 20 supervised sessions.

Participants assigned to aerobic exercise completed a 4-week programme of continuous, body-weight supported, moderate-intensity aerobic treadmill training which represented the standard, safe approach for subacute stroke rehabilitation at the time when the trial was designed. Sessions consisted of a brief warm-up followed by approximately 25 min of active training. Exercise intensity was prescribed at 55%−65% of age-predicted maximal heart rate, estimated using the predefined pragmatic formula 180 minus age and reduced by 10 beats per minutes in participants that received β-blockers. This approach was chosen as a safety-oriented and clinically feasible intensity prescription for moderately to severely affected patients in early subacute inpatient rehabilitation (Flöel et al., 2014). Training intensity was monitored *via* continuous heart-rate sensor tracking and adjusted dynamically if needed by reducing the bodyweight support, increasing the belt speed, or adjusting the inclination. Parachute harnesses were applied for safety reasons to all participants in the training group to prevent from falls.

Control group participants received progressive muscle relaxation based on the Jacobson method. Session frequency (5 times per week over 4 week period) and duration (50 min session length with 25 min core intervention time) corresponded to the aerobic intervention. The protocol emphasized structured contraction and release of muscle groups under supervision, focusing on relaxation and body awareness while avoiding relevant cardiovascular activation. Heart rate were monitored and ratings of perceived exertion assessed as well to confirm the absence of cardiovascular stress. Intervention delivery and outcome assessment were performed by different personnel to reduce potential performance and detection bias.

### Outcomes and biomarker assessment

Blinded assessors performed evaluations at baseline, immediately after the 4-week intervention period (V1), and at three (V2) and 6 months (V3) post-stroke. Baseline data included demographic variables, medical history, stroke etiology, and clinical presentation. Functional independence was measured using the Barthel Index. Gait performance was assessed *via* maximal walking speed over 10 m, with participants instructed to walk as fast as safely possible.

Depressive symptoms were quantified using the Center for Epidemiologic Studies Depression Scale (CES-D), a validated 20-item instrument for use in stroke populations (Parikh et al., 1989). The CES-D sum score ranges from 0 to 60, with higher values indicating greater depressive symptom burden. A separate response-validity criterion is integrated into the questionnaire and is derived from four questions for which the order of the responses is flipped. This criterion was applied to identify invalid CES-D questionnaires; this criterion was distinct from the CES-D sum score and values ≤ -28 were considered invalid and therefore the sum score of those participants was treated as missing. Depression has previously been associated with reduced circulating BDNF concentrations (Mojtabavi et al., 2022).

At each study visit, fasting venous blood samples were obtained at standardized time points. Blood was collected into tubes without anticoagulant for serum preparation. Samples were allowed to clot and were subsequently centrifuged (10 min with centrifugation speed at 2500 × g). The resulting serum was aliquoted and stored at −80°C until batch analysis. Samples underwent one freeze–thaw cycle before analysis.

Serum mature brain-derived neurotrophic factor (mBDNF) concentrations were measured using the MILLIPLEX^®^ MAP Human Neurodegenerative Disease Magnetic Bead Panel 3 (HNDG3MAG-36K; Merck-Millipore). The assay is a Luminex^®^ xMAP^®^-based multiplex particle immunoassay, and measurements were acquired using a Luminex^®^ 200^TM^ instrument. According to the manufacturer, the BDNF immunogen comprised amino acids Arg128–Arg247 of human BDNF, corresponding to the mature BDNF region. The present assay therefore measured mature BDNF rather than total BDNF. ProBDNF was not measured separately, and the mature-BDNF-to-proBDNF ratio could not be determined. The assay was performed according to the manufacturer's instructions.

All samples were analysed in three batches with one measurement per sample. Calibration and quality-control procedures were applied for each batch. The manufacturer's protocol specifies human serum and plasma as suitable sample matrices and recommends a minimum clotting time of 30 minutes, centrifugation at 1000 × g for 10 minutes, 1:100 sample dilution, and avoidance of more than two freeze–thaw cycles. BDNF concentrations are reported in ng/mL. Platelet counts were included as a covariate because platelet number and platelet release during coagulation may influence serum mBDNF concentrations.

### Statistical analyses

Continuous variables are presented as mean (standard deviation) or median [interquartile range], as appropriate. Categorical variables are presented as counts and percentages. Associations between platelet counts and serum BDNF concentrations were examined using Pearson correlation coefficients.

Serum BDNF served as the primary endpoint of this biomarker-focused analysis. The principal analysis assessed longitudinal treatment effects on serum BDNF using linear mixed-effects models for repeated measures. Serum BDNF was used as the dependent variable, with treatment group, time point, and their interaction included as fixed effects. Models were adjusted for baseline serum BDNF and further controlled for age, sex, stroke severity quantified by the National Institutes of Health Stroke Scale, platelet count, baseline CES-D score, and pre-stroke smoking status. Platelet count was incorporated because of its contribution to circulating BDNF concentrations. To account for multicentre recruitment, study center was included as a covariate. Because model convergence was unstable with center specified as a random intercept, centers were grouped into three predefined categories for analysis.

Incomplete observations including invalid CES-D sum scores were addressed using multiple imputation by chained equations under a missing-at-random assumption, implemented with the “mice” package in R. We generated 1,000 imputed datasets with 5 iterations per imputation. The predictor matrix was prespecified to reflect plausible associations and the estimates were pooled using Rubin's rule. Descriptive analyses are based on observed data, whereas regression analyses were performed using the imputed datasets unless stated otherwise. A complete-case analysis was conducted as sensitivity analysis.

Although the biomarker analyses were prespecified in the statistical analysis plan of the PHYS-STROKE trial, they were considered exploratory (Statistical analysis plan of the PHYS-STROKE trial can be found at https://doi.org/10.6084/m9.figshare.5375026.v1; Nave et al., 2013) therefore, correction for multiple testing was not applied. Exploratory analyses evaluated potential effect modification by sex through inclusion of a treatment-by-sex interaction term. A separate exploratory model incorporated a treatment-by-baseline CES-D interaction to investigate whether depressive symptoms moderated treatment effects. The number of completed intervention sessions was examined as a continuous predictor in an additional exploratory model independent of treatment allocation. Longitudinal analyses describing overall serum BDNF trajectories irrespective of treatment assignment as well as associations of baseline BDNF with maximal walking speed are provided in the Supplement.

All statistical analyses were performed using R Project for Statistical Computing (RRID:SCR_001905) with the packages tidyverse (RRID:SCR_019186) and mice (RRID:SCR_026363; van Buuren and Groothuis-Oudshoorn, 2011). Analysis code will be made publicly available upon publication.

## Results

Of 200 patients randomized in the PHYS-STROKE trial, serum BDNF measurements at baseline and at least one follow-up time point were available for 156 participants. Complete serum BDNF data across all time points were available for 118 participants. Reasons for missing blood samples included early discharge, withdrawal of consent, loss to follow-up, and logistical constraints during follow-up visits. Participant flow for the biomarker assessments is shown in Figure 1. Descriptive results are based on observed data; regression analyses used multiple imputation as specified in the Methods.

**Figure 1:**
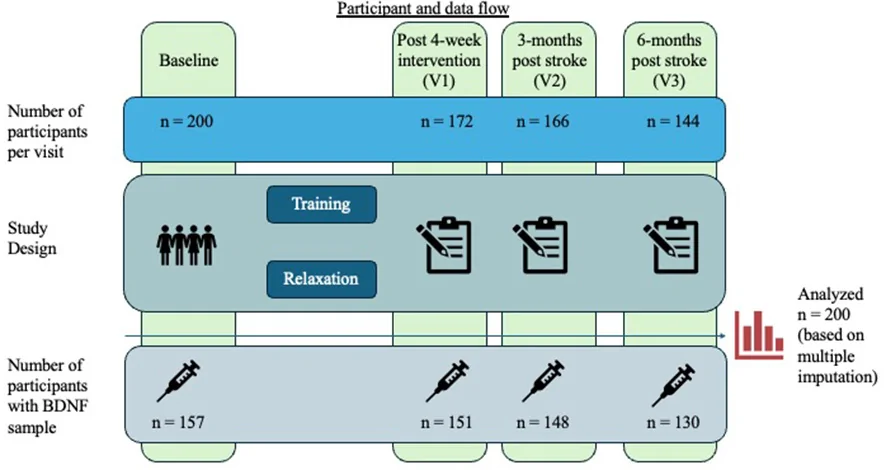
Patient and data flow.

Baseline characteristics are summarized in Table 2. Participants were moderately to severely affected, with a median National Institutes of Health Stroke Scale score of 8 [IQR 5–12] and a median Barthel Index of 50 [35–60]. Mean serum BDNF concentration at baseline was 17.2 ng/mL (SD 13.2). Mild depressive symptoms were common at baseline, with a median Center for Epidemiologic Studies Depression Scale score of 10 [6–14]. Despite randomization, baseline serum BDNF concentrations were higher in the aerobic training group than in the relaxation group, and stroke severity was slightly greater in the aerobic training group. At baseline, serum BDNF showed a weak positive association with stroke severity (*r* = 0.17, *p* = 0.031) and a weak negative association with depressive symptoms (*r* = −0.21, *p* = 0.021). The correlation between platelet count and serum BDNF was weak and did not reach statistical significance (*r* = 0.14, *p* = 0.087).

**Table 2:** Baseline characteristics of all participants and separated per intervention group.

Baseline characteristics	Training (*n* = 105)	Relaxation (*n* = 95)	All (*n* = 200)
Age, mean (SD)	69 [12]	70 [11]	69 [12]
Female, *n* (%)	45 (43)	36 (38)	81 (41)
Days post stroke, mean (sd)	26 (12)	27 (17)	27 (15)
Ischemic stroke, *n* (%)	91 (87)	90 (95)	181 (91)
Treatment with alteplase, *n* (%)^#^	34 (37)	27 (30)	61 (34)
Previous stroke, *n* (%)	27 (27)	27 (28)	54 (27)
Diabetes mellitus, *n* (%)	32 (31)	31 (33)	63 (32)
Cardiovascular disease, *n* (%)	11 (11)	18 (19)	29 (15)
Hypercholesterolaemia, *n* (%)	43 (41)	37 (39)	80 (40)
Current smoker, *n* (%)	31 (30)	32 (34)	63 (32)
NIHSS, median [IQR]	9 [5–12]	7 [5–11]	8 [5–12]
Maximal walking speed, m/s, mean (sd)	0.4 (0.4)	0.5 (0.4)	0.4 (0.4)
Barthel Index, median [IQR]	50 [35–60]	55 [35–65]	50 [35–60]
CES-D, median [IQR]	10 [6–16] (*n* = 85)	11 [6–14] (*n* = 72)	10 [6–14] (*n* = 159)
BDNF, ng/ml, mean (sd)	19.7 (16) (*n* = 83)	14.3 (8.2) (*n* = 74)	17.2 (13.2) (*n* = 157)
Thrombocytes, no/ml	279.5 (89.8) (*n* = 102)	282.3 (106.9) (*n* = 94)	280.8 (98.1) (*n* = 196)

#refers only to patients with ischemic stroke

The aerobic training group completed a mean of 16 (SD 6) sessions [core duration: 21 (SD 4) min/session], while the relaxation group completed 17 (SD 5) sessions [core duration: 24 (SD 3) min/session]. Fewer than 15 total sessions were completed by 17% (*n* =18) of aerobic training participants and 12% (*n* = 11) of relaxation participants. Primary reasons for early termination across both groups were transfer to non-participating facilities (*n* = 4 per group) or serious adverse events (aerobic: *n* = 5; relaxation: *n* = 3). Mean pre-to-post-session heart rate increase was 15 (SD 9) bpm for aerobic training vs. −2 (SD 3) bpm for relaxation. Participants achieved the predefined target intensity in a median of 70% [IQR 23–100] of training sessions, with substantial between-participant variability.

### Effect of treatment allocation on serum BDNF

Serum BDNF increased modestly over time across the cohort, with most of the increase occurring between baseline and 3 months post-stroke. In the primary adjusted analysis, serum BDNF concentrations at 6 months post-stroke did not differ between participants allocated to aerobic training and those allocated to relaxation therapy (adjusted mean difference −1.15 ng/mL, SE 3.42; *p* = 0.738; Table 3). Although baseline serum BDNF concentrations were higher in the aerobic training group, concentrations converged over time, resulting in similar estimated mean levels at 6 months post-stroke (training: 24.6 ng/mL, 95% CI 20.2–29.0; relaxation: 24.3 ng/mL, 95% CI 19.8–28.8; Figure 2).

**Table 3:** Progression of BDNF in both groups and per sex.

Time	Training			Relaxation		
	Male, ng/ml (95% CI)	Female, ng/ml (95%CI)	*p*-value	Male, ng/ml (95% CI)	Female, ng/ml (95%CI)	*p*-value
Baseline	19.8 (16.2–23.5)	16.0 (8.9–23.1)	0.334	12.9 (9.4–16.3)	15.8 (11.1–20.5)	0.331
Post-intervention (V1)	16.7 (13.5–20.0)	17.0 (10.6–23.4)	0.935	17.8 (14.7–21.0)	22.5 (18.3–26.7)	0.078
3-months post stroke (V2)	24.0 (18.8–29.2)	20.8 (10.6–31.0)	0.568	22.1 (17.1–23.1)	20.7 (13.7–27.7)	0.748
6-months post stroke (V3)	23.5 (17.9–29.2)	14.7 (3.4–26.1)	0.148	20.9 (15.7–26.2)	31.0 (22.9–39.0)	**0.039**
V1–Baseline	−0.1 (−3.3 to 3.0)	3.9 (−6.3 to 14.2)	0.935	1.0 (−2.2 to 4.1)	5.6 (1.4–9.8)	0.078
V2–V1	7.3 (2.1–12.5)	3.8 (−6.5 to 14.1)	0.535	4.2 (−0.7 to 9.3)	−1.8 (−8.7 to 5.2)	0.167
V3–V2	−0.5 (−6.6 to 5.6)	−6.1 (−18.0 to 5.9)	0.382	−1.2 (−6.8 to 4.4)	10.2 (1.9–18.6)	**0.024**

Interaction effect of treatment allocation and sex on Serum BDNF levels at each study visit and the change between each visit. Values based on multiple imputation with chained equations. Bold values indicate statistical significant interactions between group allocation and gender against a Type–1 error rate of 0.05.

**Figure 2:**
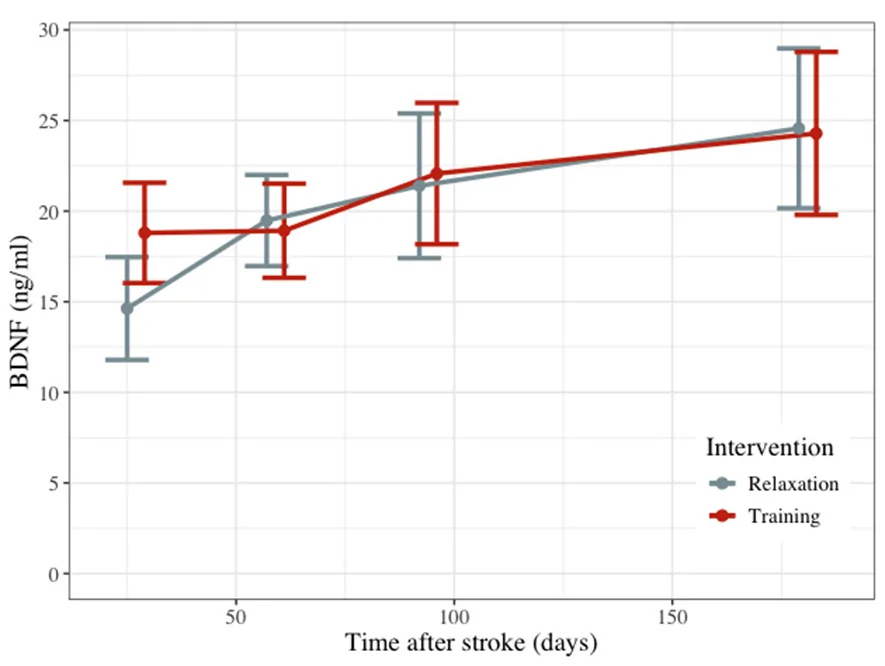
Serum BDNF progression over time in both intervention groups. Mean serum BDNF levels (ng/mL) with 95% confidence intervals from baseline through 6 months post-stroke in the aerobic training (red) and relaxation therapy (grey) groups.

Because the biological effect of a 4-week exercise intervention may be most apparent immediately after the intervention, we also examined post-intervention serum BDNF levels at V1. No relevant between-group difference was observed at this time point. Thus, the absence of a between-group difference was not restricted to the 6-month follow-up but was also evident at the assessment immediately after the intervention period. Sensitivity analyses using complete cases and change scores from baseline yielded consistent results, with no evidence of a between-group difference in serum BDNF at follow-up.

### Exploratory effect modification analyses

Exploratory analyses did not provide robust evidence that the effect of treatment allocation on serum BDNF differed by depressive symptom burden. Additional *post hoc* analyses showed no indication that baseline depressive symptom severity influenced the longitudinal treatment effect on serum BDNF (Table S1 and Figure S1; all *p* values > 0.05). Sex-stratified analyses suggested a nominal difference in 6-month serum BDNF concentrations between women and men in the relaxation group (females: 31.0 ng/mL, 95% CI 22.9–39.0; males: 20.9 ng/mL, 95% CI 15.7–26.2; *p* = 0.039), whereas no comparable difference was observed in the aerobic training group (females: 14.7 ng/mL, 95% CI 3.4–26.1; males: 23.5 ng/mL, 95% CI 17.9–29.2; *p* = 0.148, Figure S2). Given the exploratory nature of this analysis, the lack of correction for multiple testing, and the small subgroup sizes, this finding should be interpreted cautiously.

Within the aerobic training group, no association was observed between the number of completed training sessions and changes in serum BDNF during the intervention period or over subsequent follow-up intervals. Estimates were small and imprecise, with confidence intervals spanning zero. Repeated-measures analyses showed a modest increase in serum BDNF over time across the cohort, with substantial inter-individual variability and no clear differences by time-from-stroke category at inclusion (Figures S3 and Figures S4). Also, we could not identify any association of serum BDNF measured at baseline with maximal walking speed as clinical outcome. These analyses are presented for descriptive purposes and are shown in the Supplementary Material (Figure S5).

## Discussion

In this pre-specified secondary biomarker analysis of the PHYS-STROKE trial, 4 weeks of moderate-intensity treadmill-based aerobic training initiated in the early subacute phase after stroke was not associated with higher serum BDNF concentrations compared with relaxation therapy. Serum BDNF increased modestly over time across the cohort, but no relevant between-group difference was observed either immediately after the intervention period or at 6 months post-stroke. These findings suggest that this clinically feasible aerobic exercise intervention did not induce a robust sustained peripheral BDNF response in a heterogeneous, moderately to severely affected subacute stroke population.

The absence of a between-group difference is consistent with previous randomized trials and systematic reviews reporting small, inconsistent, or absent effects of aerobic exercise on peripheral BDNF after stroke (Ashcroft et al., 2022; Limaye et al., 2021). Although aerobic exercise can increase circulating BDNF in healthy individuals, this effect may not generalize uniformly to patients in the early phase after stroke (Müller et al., 2020; De Las Heras et al., 2025). Stroke-related systemic inflammation, autonomic dysregulation, altered platelet dynamics, comorbidities, medication and tobacco use, and reduced exercise capacity may all influence circulating BDNF concentrations and attenuate exercise-related responses (Rasmussen et al., 2009; Pareja-Galeano et al., 2015; Ehrhardt et al., 2024; Shafiee et al., 2024). In addition, spontaneous biological recovery processes may contribute to changes in serum BDNF over time, making it difficult to separate intervention-specific effects from the broader post-stroke recovery trajectory (Berretta et al., 2014).

The timing of blood sampling is important when interpreting these findings. Recent studies in early subacute stroke have reported no significant acute or chronic serum BDNF response after cardiovascular exercise, whereas other studies and meta-analyses suggest that acute responses may be more detectable after higher-intensity exercise (De Las Heras et al., 2025). Because the present study did not obtain blood samples during or immediately after individual exercise sessions, transient within-session or early post-exercise responses cannot be excluded.

The pragmatic nature of the intervention also warrants consideration. Recent evidence suggests that exercise intensity is an important determinant of acute BDNF responses after stroke, with more consistent increases reported after high-intensity aerobic exercise than after low- or moderate-intensity protocols. A recent network meta-analysis in adults similarly reported a stronger acute peripheral BDNF response after high-intensity interval training than after moderate-intensity continuous training, although uncertainty remained regarding chronic effects (Rodríguez-Gutiérrez et al., 2024). The intervention in PHYS-STROKE was designed as a safety-oriented, moderate-intensity programme for patients with moderate to severe impairment in the early subacute phase and was not intended to maximize acute neurotrophin release. Therefore, our findings should be interpreted as evidence against a robust sustained serum BDNF response to this clinically pragmatic intervention, rather than as evidence against all possible effects of higher-intensity or more individualized exercise.

Exploratory analyses did not provide robust evidence that depressive symptoms or intervention dose modified the effect of aerobic training on serum BDNF. A nominal sex-related subgroup finding was observed in the relaxation group, with higher 6-month BDNF concentrations among women than men, whereas no comparable pattern was observed in the aerobic training group. Given the exploratory nature of this analysis, small subgroup sizes, lack of correction for multiple testing, and absence of a clear treatment-specific biological rationale, this finding should be interpreted cautiously and may reflect chance. It should therefore be regarded as hypothesis-generating only and should not inform stratified rehabilitation approaches without replication in adequately powered studies.

Several limitations need to be considered. First, despite randomization in the parent trial, baseline serum BDNF concentrations were higher in the aerobic training group than in the relaxation group, and stroke severity was slightly greater in the aerobic training group. Although all primary models adjusted for baseline BDNF, stroke severity, age, sex, platelet count, depressive symptoms, smoking status, and study center, residual bias cannot be excluded. The convergence of serum BDNF levels over time may partly reflect regression to the mean rather than a biological treatment-related pattern. Second, missing biomarker data were common, reflecting the practical challenges of repeated blood sampling in subacute stroke trials with longitudinal follow-up (Tyson et al., 2015). We addressed incomplete observations using multiple imputation and performed complete-case sensitivity analyses, which yielded consistent results. However, the missing-at-random assumption cannot be verified, and attrition may have introduced bias if participants with available follow-up samples differed systematically from those without complete biomarker data. Third, serum BDNF is an imperfect peripheral marker of neuroplasticity. Circulating BDNF is strongly influenced by platelet storage and release and may be affected by metabolic, inflammatory, neuroendocrine, and pharmacological factors (Béjot et al., 2011; Naegelin et al., 2018). Also, the assay and blood matrix also limit comparability with newer studies. Serum and plasma BDNF are not interchangeable measures, and recent methodological reviews have emphasized substantial variability in the reporting of clotting, centrifugation, temperature, anticoagulant use, and platelet content in stroke studies (Bevilacqua et al., 2026; Ng, 2025). While platelet count was included as a covariate, serum BDNF cannot be interpreted as a direct surrogate of central nervous system BDNF signaling. The lack of a peripheral serum BDNF response therefore does not exclude exercise-related effects on central neuroplasticity or functional recovery through other biological pathways. Fourth, the PHYS-STROKE trial was powered for clinical outcomes rather than biomarker subgroup analyses. The present analysis was pre-specified but exploratory, and subgroup findings should be interpreted with caution. We also did not assess BDNF Val66Met genotype, which may influence activity-dependent BDNF release and could contribute to inter-individual variability in biomarker responses (Dinoff et al., 2016; Berretta et al., 2014; Rodier et al., 2015). Fifth, the broad inclusion window of 5–45 days after stroke captured patients at different stages of early recovery, which may have increased biological heterogeneity and the intervention intensity and length was potentially inadequate to induce real BDNF changes. When the PHYS-STROKE trial was designed, moderate-intensity continuous training represented the standard, safe approach for subacute stroke rehabilitation. Newer evidence suggests that BDNF release in humans is intensity-dependent, often requiring higher physiological strain (e.g., high-intensity interval training) than the moderate target heart rates utilized in this protocol. Additionally, the 4-week intervention period was dictated by inpatient care durations, while the 6-month follow-up aimed to track broader post-stroke recovery trajectories rather than persistent direct intervention effects. Finally, although minor fluctuations were observed in the relaxation group, overall study findings were neutral. Given that secondary biomarkers such as cortisol demonstrated no group differences, these isolated signals should be interpreted cautiously and likely reflect non-specific baseline recovery within a small sub-sample rather than a distinct biological effect of relaxation therapy.

## Conclusion

In conclusion, 4 weeks of moderate-intensity aerobic training in the early subacute phase after stroke did not increase serum mature BDNF compared with relaxation therapy. These findings argue against a robust sustained peripheral BDNF response to this intervention under clinically feasible inpatient rehabilitation conditions. They do not exclude acute, higher-intensity, genotype-dependent, or subgroup-specific neurotrophin responses, and future studies should consider more temporally dense biomarker sampling, individualized exercise intensity prescription, and biological stratification when investigating exercise-induced neuroplasticity after stroke.

## Data Availability

Analysis scripts unique to the current manuscript are freely accessible on GitHub: https://github.com/Funkstille1011/BDNF. Anonymized individual-level patient data can be accessed under data-sharing agreements from the corresponding author.

## References

[B1] Alcantara C. C. García-Salazar L. F. Silva-Couto M. A. Santos G. L. Reisman D. S. Russo T. L. (2018). Post-stroke BDNF concentration changes following physical exercise: a systematic review. Front. Neurol. 9:637. doi: 10.3389/fneur.2018.00637 30210424 PMC6121011

[B2] Ashcroft S. K. Ironside D. D. Johnson L. Kuys S. S. Thompson-Butel A. G. (2022). Effect of exercise on brain-derived neurotrophic factor in stroke survivors: a systematic review and meta-analysis. Stroke. 53, 3706–16. doi: 10.1161/STROKEAHA.122.039919 36278401

[B3] Berretta A. Tzeng Y. C. Clarkson A. N. (2014). Post-stroke recovery: the role of activity-dependent release of brain-derived neurotrophic factor. Expert Rev. Neurother. 14, 1335–1344. doi: 10.1586/14737175.2014.969242 25319267

[B4] Béjot Y. Mossiat C. Giroud M. Prigent-Tessier A. Marie C. (2011). Circulating and Brain BDNF levels in stroke rats: relevance to clinical studies. PLoS ONE. 6:e29405. doi: 10.1371/journal.pone.0029405 22195050 PMC3241711

[B5] Bevilacqua E. Cacciante L. Burgio F. . (2026). Reporting of pre-analytical variables affecting the determination of brain-derived neurotrophic factor (BDNF) in patients with stroke: a scoping review. Clin. Chem. Lab. Med. CCLM. 64, 1722–1733. doi: 10.1515/cclm-2026-0277 42034454

[B6] Billinger S. A. Arena R. Bernhardt J. Eng J. J. Franklin B. A. Johnson C. M. . (2014). Physical activity and exercise recommendations for stroke survivors: a statement for healthcare professionals from the American Heart Association/American Stroke Association. Stroke. 45, 2532–2553. doi: 10.1161/STR.0000000000000022 24846875

[B7] Boukhatem I. Fleury S. Jourdi G. Lordkipanidzé M. (2024). The intriguing role of platelets as custodians of brain-derived neurotrophic factor. Res. Pract. Thromb. Haemost. 8:102398. doi: 10.1016/j.rpth.2024.102398 38706782 PMC11066552

[B8] De Las Heras B. Rodrigues L. Cristini J. . (2025). Investigating the acute and chronic effects of cardiovascular exercise on brain-derived neurotrophic factor in early subacute stroke. Neurorehabil. Neural Repair. 39, 653–665. doi: 10.1177/154596832513421 40462267 PMC12318165

[B9] Dinoff A. Herrmann N. Swardfager W. Liu C. S. Sherman C. Chan S. . (2016). The effect of exercise training on resting concentrations of peripheral brain-derived neurotrophic factor (BDNF): a meta-analysis. PLoS ONE. 11:e0163037. doi: 10.1371/journal.pone.0163037 PMC5033477 27658238

[B10] Dromerick A. W. Geed S. Barth J. Brady K. Giannetti M. L. Mitchell A. . (2021). Critical period after stroke study (CPASS): a phase II clinical trial testing an optimal time for motor recovery after stroke in humans. Proc. Natl. Acad. Sci. 118:e2026676118. doi: 10.1073/pnas.2026676118 PMC8488696 34544853

[B11] Ehrhardt M. Schreiber S. Duderstadt Y. . (2024). Circadian rhythm of brain-derived neurotrophic factor in serum and plasma. Exp. Physiol. 109, 1755–1767. doi: 10.1113/EP091671 PMC11442779 39105714

[B12] Flöel A. Werner C. Grittner U. Hesse S. Jöbges M. Knauss J. . (2014). Physical fitness training in subacute stroke (PHYS-STROKE)–study protocol for a randomised controlled trial. Trails. 15:45. doi: 10.1186/1745-6215-15-45 24491065 PMC3922602

[B13] Gejl A. K. Enevold C. Bugge A. Andersen M. S. Nielsen C. H. Andersen L. B. (2019). Associations between serum and plasma brain-derived neurotrophic factor and influence of storage time and centrifugation strategy. Sci. Rep. 9:9655. doi: 10.1038/s41598-019-45976-5 31273250 PMC6609657

[B14] Li S. (2023). Stroke recovery is, a journey: prediction and potentials of motor recovery after a stroke from a practical perspective. Life. 13:2061. doi: 10.3390/life13102061 PMC10608684 37895442

[B15] Li X. He Y. Wang D. Rezaei M. J. (2024). Stroke rehabilitation: from diagnosis to therapy. Front. Neurol. 15:1402729. doi: 10.3389/fneur.2024.1402729 39193145 PMC11347453

[B16] Limaye N. S. Carvalho L. B. Kramer S. (2021). Effects of aerobic exercise on serum biomarkers of neuroplasticity and brain repair in stroke: a systematic review. Arch. Phys. Med. Rehabil. 102, 1633–1644. doi: 10.1016/j.apmr.2021.04.010 33992633

[B17] MacKay-Lyons M Billinger SA Eng JJ Dromerick A. Giacomantonio N. Hafer-Macko C. . (2020). Aerobic exercise recommendations to optimize best practices in care after stroke: AEROBICS 2019 update. Phys. Ther. 100:149–156. doi: 10.1093/ptj/pzz153 31596465 PMC8204880

[B18] Maguire C. Betschart M. Pohl J. Primani F. Taeymans J. Hund-Georgiadis M. (2023). Effects of moderate-intensity aerobic exercise on serum BDNF and motor learning in the upper-limb in patients after chronic-stroke: a randomized, controlled feasibility study with embedded health economic evaluation. NeuroRehabilitation. 52, 485–506. doi: 10.3233/NRE-220239 36806518

[B19] Mang C. S. Campbell K. L. Ross C. J. D. Boyd L. A. (2013). Promoting neuroplasticity for motor rehabilitation after stroke: considering the effects of aerobic exercise and genetic variation on brain-derived neurotrophic factor. Phys. Ther. 93, 1707–16. doi: 10.2522/ptj.20130053 PMC3870490 23907078

[B20] Medical University of Graz. Randomizer. Available online at: http://www.randomizer.at/ (Accessed July 01 2024)..

[B21] Mojtabavi H. Shaka Z. Momtazmanesh S. Ajdari A. Rezaei N. (2022). Circulating brain-derived neurotrophic factor as a potential biomarker in stroke: a systematic review and meta-analysis. J. Transl. Med. 20:126. doi: 10.1186/s12967-022-03312-y PMC8919648 35287688

[B22] Müller P. Duderstadt Y. Lessmann V. Müller N. G. (2020). Lactate and BDNF: key mediators of exercise induced neuroplasticity? J. Clin. Med. 9:1136. doi: 10.3390/jcm9041136 PMC7230639 32326586

[B23] Naegelin Y. Dingsdale H. Säuberli K. Schädelin S. Kappos L. Barde Y. A. (2018). Measuring and validating the levels of brain-derived neurotrophic factor in human serum. eneuro 5. doi: 10.1523/ENEURO.0419-17.2018 PMC5898630 29662942

[B24] Nave A. H. Kröber J. M. Brunecker P. Fiebach J. B. List J. Grittner U. . (2013). Biomarkers and perfusion-training-induced changes after stroke (BAPTISe): protocol of an observational study accompanying a randomized controlled trial. BMC Neurol. 13:197. doi: 10.1186/1471-2377-13-197 PMC3870989 24330706

[B25] Nave A. H. Rackoll T. Grittner U. Bläsing H. Gorsler A. Nabavi D. G. . (2019). Physical fitness training in patients with subacute stroke (PHYS-STROKE): multicentre, randomised controlled, endpoint blinded trial. BMJ. Published online, l5101. doi: 10.1136/bmj.l5101 PMC6749174 31533934

[B26] Ng T. K. (2025). Clarifying blood-based brain-derived neurotrophic factor measurement in Alzheimer's disease research: a call for pre-analytic standardization. J. Alzheimers. Dis. Rep. 9. doi: 10.1177/25424823251399060 PMC12644425 41306381

[B27] Pareja-Galeano H. Alis R. Sanchis-Gomar F. . (2015). Methodological considerations to determine the effect of exercise on brain-derived neurotrophic factor levels. Clin. Biochem. 48, 162–166. doi: 10.1016/j.clinbiochem.2014.11.013 25464018

[B28] Parikh R. M. Eden D. T. Price T. R. Robinson R. G. (1989). The sensitivity and specificity of the center for epidemiologic studies depression scale in screening for post-stroke depression. Int. J. Psychiatry Med. 18, 169–181. doi: 10.2190/BH75-EUYA-4FM1-J7QA 3170080

[B29] Rasmussen P. Brassard P. Adser H. Pedersen M. V. Leick L. Hart E. Evidence for a release of brain-derived neurotrophic factor from the brain during exercise: Brain-derived neurotrophic factor release during exercise. Exp. Physiol 2009; 94:1062–1069. doi: 10.1113/expphysiol.2009.048512. 19666694

[B30] Reichardt L. F. (2006). Neurotrophin-regulated signalling pathways. Philos. Trans. R Soc. B Biol. Sci. 361, 1545–64. doi: 10.1098/rstb.2006.1894 16939974 PMC1664664

[B31] Rodier M. Quirié A. Prigent-Tessier A. Béjot Y. Jacquin A. Mossiat C. . (2015). Relevance of post-stroke circulating BDNF levels as a prognostic biomarker of stroke outcome: impact of rt-PA treatment. PLoS ONE. 10:e0140668. doi: 10.1371/journal.pone.0140668 26469350 PMC4607484

[B32] Rodríguez-Gutiérrez E. Torres-Costoso A. Saz-Lara A. . (2024). Effectiveness of high-intensity interval training on peripheral brain-derived neurotrophic factor in adults: A systematic review and network meta-analysis. Scand. J. Med. Sci. Sports. 34:e14496. doi: 10.1111/sms.14496 37728896

[B33] Shafiee A. Beiky M. Mohammadi I. Rajai S. Jafarabady K. Moradi S. . (2024). Effect of smoking on brain-derived neurotrophic factor (BDNF) blood levels: a systematic review and meta-analysis. J. Affect. Disord. 349, 525–533. doi: 10.1016/j.jad.2024.01.082 38199418

[B34] Tyson S. F. Thomas N. Vail A. Tyrrell P. (2015). Recruiting to inpatient-based rehabilitation trials: lessons learned. Trails. 16, 2–5. doi: 10.1186/s13063-015-0588-2 PMC4350304 25886846

[B35] >van Buuren S. Groothuis-Oudshoorn K. (2011). Mice: multivariate imputation by chained equations in R. J. Stat. Soft. 45, 1–67. doi: 10.18637/jss.v045.i03

