# Supplementary material for: Serum BDNF in subacute stroke: effects of a four-week aerobic exercise intervention in the PHYS-STROKE trial
Source: Front Stroke. 2026 Sep 15;5:1897859. doi: 10.3389/fstro.2026.1897859 (PMC13619496; doi:10.3389/fstro.2026.1897859)
Supplement: Supplementary file 1 [file Supplementary_file_1.docx]

**Serum BDNF in Subacute Stroke: Effects of a Four-Week Aerobic Exercise Intervention**

Torsten Rackoll, Lea Doppelbauer, Konrad Neumann and Alexander Heinrich Nave

Supplemental Appendix

Contents

[Table S1	2](#_Toc237009824)

[Figure S1	3](#_Toc237009825)

[Figure S2	4](#_Toc237009826)

[Dose-response relationship	5](#_Toc237009827)

[Figure S3	6](#_Toc237009828)

[Figure S4	7](#_Toc237009829)

[Figure S5	8](#_Toc237009830)

# Table S1

Association of the interaction of CES-D and group allocation on serum BDNF

|  | Intercept Relaxation,  mean (95% CI) | Slope Relaxation,  mean (95% CI) | Intercept Training,  mean (95% CI) | Slope Training,  mean (95%CI) | p value for the intercept | p-value for the slope |
| --- | --- | --- | --- | --- | --- | --- |
| Baseline | 15,72 (9,63 - 21,80) | -0,17 (-0,71 - 0,38) | 23,88 (18,12 - 29,65) | -0,43 (-0,89 - 0,03) | 0,047 | 0,445 |
| V1 | 20,92 (15,01 - 26,83) | -0,12 (-0,67 - 0,43) | 21,16 (15,77 - 26,54) | -0,24 (-0,65 - 0,17) | 0,950 | 0,713 |
| V2 | 19,40 (11,01 -27,80) | 0,21 (-0,56 - 0,98) | 21,63 (13,30 - 29,97) | 0,05 (-0,62 - 0,71) | 0,699 | 0,737 |
| V3 | 26,22 (16,47 - 35,97) | -0,13 (-1,05 - 0,79) | 22,73 (13,07 - 32,38) | 0,11 (-0,63 - 0,85) | 0,579 | 0,653 |
| V1-BL | 4,07 (-1,85 - 9,99) | -0,12 (-0,67 - 0,43) | 4,31 (-1,00 - 9,61) | -0,24 (-0,65 - 0,17) | 0,950 | 0,713 |
| V2-BL | 2,56 (-5,84 - 10,95) | 0,21 (-0,56 - 0,98) | 4,78 (-3,53 - 13,09) | 0,05 (-0,62 - 0,71) | 0,699 | 0,737 |
| V3-BL | 9,37 (-0,38 - 19,12) | -0,13 (-1,05 - 0,79) | 5,88 (-3,79 - 15,54) | 0,11 (-0,63 - 0,85) | 0,579 | 0,653 |
| V2-V1 | -1,51 (-9,96 - 6,93) | 0,33 (-0,44 - 1,10) | 0,48 (-7,99 - 8,95) | 0,28 (-0,39 - 0,95) | 0,731 | 0,923 |
| V3-V1 | 5,30 (-4,63 -15,24) | -0,01 (-0,94 - 0,92) | 1,57 (-8,23 - 11,37) | 0,34 (-0,40 - 1,08) | 0,564 | 0,513 |
| V3-V2 | 6,81 (-3,15 - 16,78) | -0,34 (-1,28 - 0,60) | 1,09 (-8,91 - 11,10) | 0,06 (-0,68 - 0,81) | 0,384 | 0,468 |

Linear regression models with the interaction of CES-D and group allocation as explanatory variable and serum BDNF as response variable. Analyses are not adjusted and were conducted without multiple imputation.

# Figure S1

Scatter plots depicting associations of baseline CES-D sum score values with serum BDNF at a) baseline, b) v1, c) v2 and d) v3 visits respectively. Data points are color-coded with red asterix showing data from participants in the training group and blue circles showing participants from the relaxation group. Respective color-coded regression lines are shown without confidence bands for better clarity. Intercept as well as slope and its respective 95% confidence intervals of respective lines are listed in Table S1.

Baseline CES-D and its associations to serum BDNF in both groups over time.

| a)   | b)   |
| --- | --- |
| c)   | d)   |

# Figure S2

Mean serum BDNF levels (ng/mL) with 95% confidence intervals from baseline through 6 months post-stroke in the aerobic training and relaxation therapy groups per sex.

# Dose-response relationship

Given that we could not find a treatment effect between patients being randomized to the training compared to the relaxation group, we aimed to elucidate whether the number of sessions received had an influence on serum BDNF progression irrespective of the treatment allocation. In an additional model, we used number of intervention sessions as an exploratory variable but could not find any association neither during the intervention period (β 1.7 ng/ml, 95% CI -4.3 to 7.8, *p* = 0.572) nor between either the post intervention visit and three months post stroke (β 0.5 ng/ml, 95% CI -9.3 to 10.2, *p* = 0.925) or between three to six months post stroke (β 2.3 ng/ml, 95% CI -8.6 to 13.2, *p* = 0.681). Analyses were similarly adjusted for confounding factors and based on imputed data similar to the main analyses.

# Figure S3

Overall progression of serum BDNF over the study period up to six months post stroke. Time points of assessment were baseline, after intervention, three months post stroke and six months post stroke. Blood draws were conducted on average on day 27 (SD 15) for the baseline visit, on day 59 (SD 32) for the post-intervention visit V1, on day 94 (SD 29) for the three months visit V2 and on day 181 (SD 8) for the six months visit V3.

# Figure S4

Serum BDNF progression per time window, in which the baseline visit was conducted, clustered into the time frames less and equal to 15 days after stroke, between 16 and 30 days and more than 30 days after stroke. Blood draws were conducted on average on day 27 (SD 15) for the baseline visit, on day 59 (SD 32) for the post-intervention visit V1, on day 94 (SD 29) for the three months visit V2 and on day 181 (SD 8) for the six months visit V3.

# Figure S5

Scatter plots depicting associations of baseline serum BDNF values with maximal walking speed at a) baseline, b) v1, c) v2 and d) v3 visits respectively. Data points are color-coded with red dots showing data from participants in the training group and blue squares showing participants from the relaxation group. Respective color-coded regression lines are shown without confidence bands for better clarity.

Baseline serum BDNF and its associations to maximal walking speed in both groups per visit.

| a)   | b)   |
| --- | --- |
| c)   | d)   |
